# Supplementary material for: Targeting epigenetic regulators to overcome drug resistance in the emerging human fungal pathogen Candida auris
Source: Nat Commun. 2025 May 20;16:4668. doi: 10.1038/s41467-025-59898-6 (PMC12092656; doi:10.1038/s41467-025-59898-6)
Supplement: Supplementary file 1 — Supplementary information [file 41467_2025_59898_MOESM1_ESM.pdf]

Supplementary information

Targeting epigenetic regulators to overcome drug resistance in the emerging human fungal pathogen *Candida auris*

Yuping Zhang[1,2#], Lingbing Zeng[1#], Xinhua Huang[2], Yuanyuan Wang[2], Guangsheng Chen[2], Munika Moses[2], Yun Zou[2], Sichu Xiong[2], Wenwen Xue[3], Yanmei Dong[4], Yueru Tian[5], Ming Guan[5], Lingfei Hu[6], Zhe Yin[6], Dongsheng Zhou[6*], Xiaotian Huang[1*], Changbin Chen[2,3*]

[1] School of Basic Medical Sciences, and the First Affiliated Hospital, Jiangxi Medical College, Nanchang University
[2] Joint Laboratory for Biomedical Research and Pharmaceutical Innovation, Unit of Pathogenic Fungal Infection & Host Immunity, Key Laboratory of Molecular Virology and Immunology, Shanghai Institute of Immunity and Infection, Chinese Academy of Sciences, Shanghai, China
[3] Nanjing Advanced Academy of Life and Health, Nanjing, China
[4] Department of Gastroenterology and Hepatology, Characteristic Medical Center of the Chinese People's Armed Police Force, Tianjin Key Laboratory of Hepatopancreatic Fiberosis and Molecular Diagnosis & Treatment, Tianjin, China
[5] Department of Laboratory Medicine, Huashan Hospital North, Shanghai Medical College, Fudan University, Shanghai, China
[6] State Key Laboratory of Pathogen and Biosecurity, Academy of Military Medical Sciences, Beijing, China

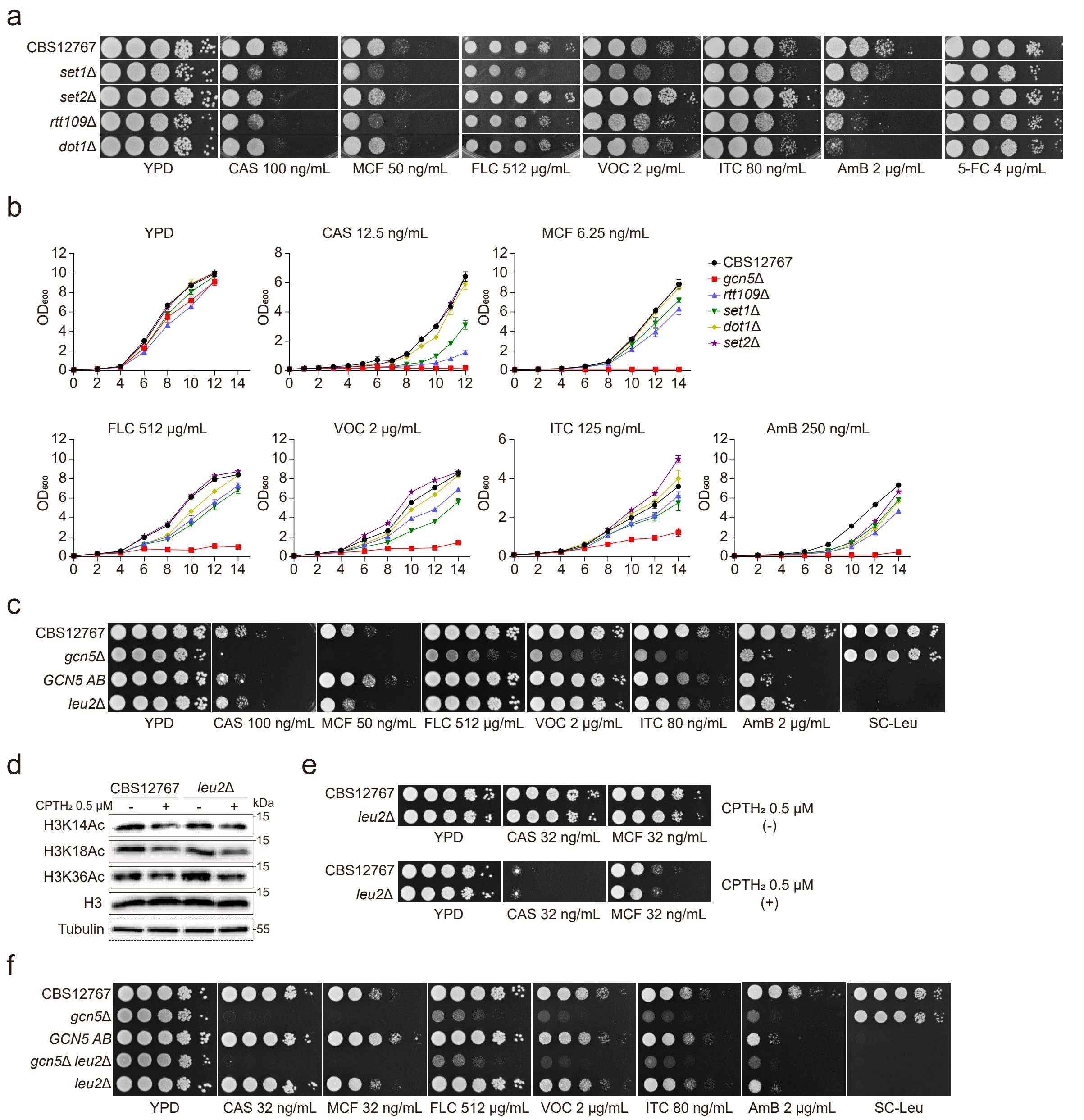

Supplementary Fig. 1 Effects of histone H3 PTMs on antifungal drug resistance in *C. auris*. **a** Spot assay comparing the antifungal drug resistance of *C. auris* CBS12767, *set1*Δ, *set2*Δ, *rtt109*Δ and *dot1*Δ strains to antifungal agents CAS, MCF, FLC, VOC, ITC, AmB, and 5-FC. **b** Growth curves of *C. auris* CBS12767, *gcn5*Δ, *set1*Δ, *set2*Δ, *rtt109*Δ, and *dot1*Δ in the presence of antifungal drugs CAS, MCF, FLC, VOC, ITC, and AmB. Data are expressed as mean ± SD and are representative of three independent experiments. **c** Spot assay comparing the growth differences of *C. auris leu2*Δ with CBS12767, *gcn5*Δ, and *GCN5 AB* strains under antifungal agents CAS, MCF, FLC, VOC, ITC, and AmB, as well as in leucine-deficient SC-Leu medium. **d** Western blot analysis of histone H3 acetylation at K14, K18, and K36 in *C. auris* CBS12767 and *leu2*Δ strains cultured with or without 0.5 μM $CPTH_2$. **e** Spot assay comparing the sensitivity of *C. auris leu2*Δ and CBS12767 strains to the antifungal agents CAS and MCF, with or without 0.5 μM $CPTH_2$. **f** Spot assay comparing the growth differences of *C. auris* CBS12767, *gcn5*Δ, *GCN5 AB*, *gcn5*Δ*leu2*Δ, and *leu2*Δ strains under treatment with the antifungal agents CAS, MCF, FLC, VOC, ITC, and AmB, as well as in leucine-deficient SC-Leu medium. Source data are provided as a Source Data file.

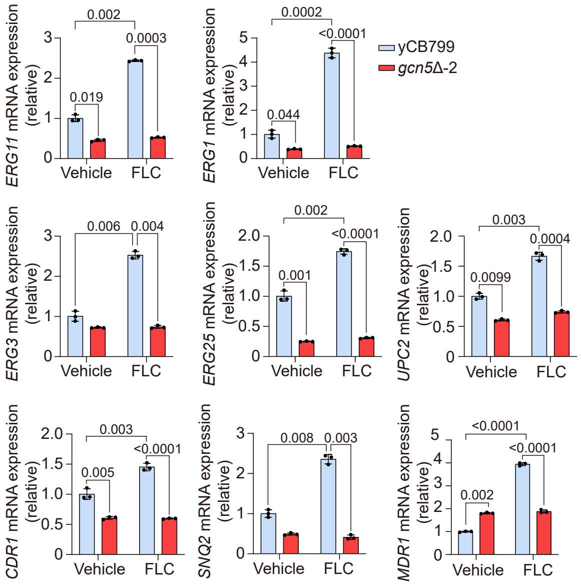

Supplementary Fig. 2 Deletion of *GCN5* leads to downregulation of genes associated with ergosterol biosynthesis pathway and drug efflux pumps in *C. auris* yCB799. qRT-PCR analysis of ergosterol biosynthesis pathway genes *ERG11*, *ERG1*, *ERG3*, *ERG25*, transcription factor *UPC2*, and drug efflux pump genes *CDR1*, *SNQ2*, and *MDR1* expression in *C. auris* yCB799 and *gcn5*Δ-2 treated with 512 μg/mL FLC or an equivalent volume of DMSO for 2 h. Data are expressed as mean ± SD and are representative of three independent experiments. Statistical significance analysis was performed using two-way ANOVA with Sidak's test. Source data are provided as a Source Data file.

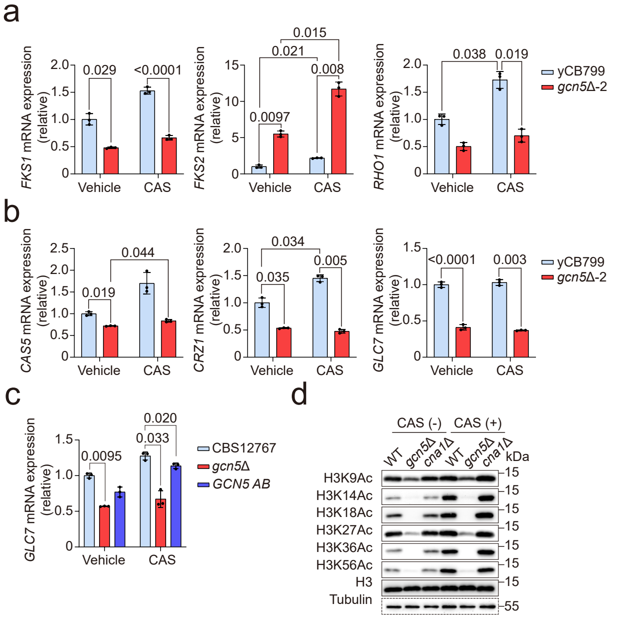

Supplementary Fig. 3 Gcn5 regulates echinocandin resistance in *C. auris* through the calcineurin pathway and transcription factor Cas5. a-b qRT-PCR analysis of *FKS1*, *FKS2* and *RHO1* (**a**), and *CAS5*, *CRZ1* and *GLC7* (**b**) gene expression in *C. auris* yCB799 and *gcn5*Δ-2 treated with 8 μg/mL CAS or an equivalent volume of DMSO for 1 h. **c** qRT-PCR analysis of *GLC7* of *C. auris* CBS12767, *gcn5*Δ and *GCN5 AB* treated with 100 ng/mL CAS or an equivalent volume of DMSO for 1 h. **d** Western blot analysis of histone H3 acetylation at various sites in *C. auris* CBS12767, *gcn5*Δ, and *cna1*Δ strains, treated with 100 ng/mL CAS or an equivalent volume of DMSO for 1 h. The experiment was independently repeated twice with consistent results. Data presented in (**a-c**) are expressed as mean ± SD and are representative of three independent experiments. Statistical significance analysis was performed using two-way ANOVA with Sidak's test. Source data are provided as a Source Data file.

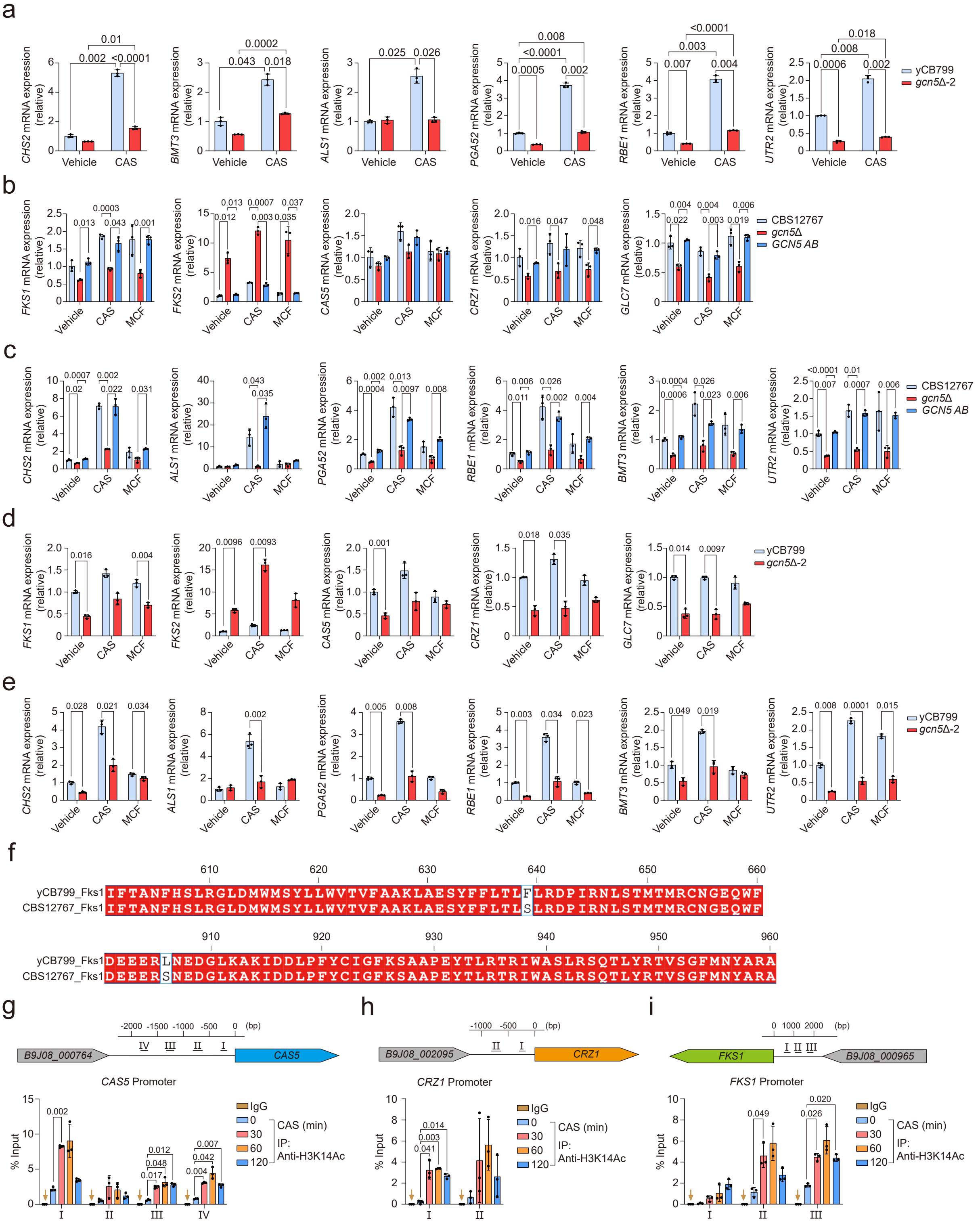

Supplementary Fig. 4 Mechanisms by which Gcn5 regulates echinocandin resistance in *C. auris*. a qRT-PCR analysis of *CHS2*, *BMT3*, *ALS1*, *PGA52*, *RBE*1, and *UTR2* gene expression in *C. auris* yCB799 and *gcn5*Δ-2 treated with 8 μg/mL CAS or an equivalent volume of DMSO for 1 h. **b-e** qRT-PCR analysis of *FKS1*, *FKS2*, *CAS5*, *CRZ1* and *GLC7* (**b**, **d**) and *CHS2*, *BMT3*, *ALS1*, *PGA52*, *RBE1*, and *UTR2* (**c**, **e**) gene expression in *C. auris* CBS12767-derived (**b**, **c**) and yCB799-derived (**d**, **e**) strains treated with 100 ng/mL (for CBS12767-derived strains) or 8 μg/mL (for yCB799-derived strains) CAS, or 50 ng/mL (for CBS12767-derived strains) or 4 μg/mL (for yCB799-derived strains) MCF, or an equivalent volume of DMSO for 1 h. **f** Sequence alignment of the Fks1

protein in *C. auris* CBS12767 and yCB799. **g-i** ChIP-qPCR analysis of histone H3K14Ac enrichment at the promoter regions of *CAS5* (**g**), *CRZ1* (**h**), and *FKS1* (**i**) genes in *C. auris* CBS12767 after treatment with 100 ng/mL CAS for 0, 30, 60, and 120 minutes. Data presented in (**a**-**e**, **g**-**h**) are expressed as mean ± SD and are representative of three independent experiments. Statistical significance analysis was performed using two-way ANOVA (**a**) or one-way ANOVA (**b**-**e**, **g**-**i**) with Sidak's test. Source data are provided as a Source Data file.

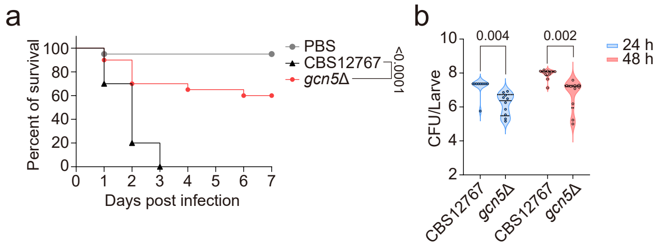

Supplementary Fig. 5 Deletion of *GCN5* attenuates the virulence of *C. auris* in *Galleria mellonella* infection model. a Survival curve of *Galleria mellonella* after infection with 1×10^6 CFU of *C. auris* CBS12767 or *gcn5*Δ mutant, or an equal volume of PBS (n=20). **b** Fungal burden in each *Galleria mellonella* larvae infected with 1×10^6 CFU of *C. auris* CBS12767 or *gcn5*Δ strains at 24 and 48 h post-infection (n=10). Data presented in (**b**) are expressed as mean ± SD. Statistical significance analysis was performed using Log-rank (Mantel-Cox) test (**a**) or two-way ANOVA with Sidak's test (**b**). Source data are provided as a Source Data file.

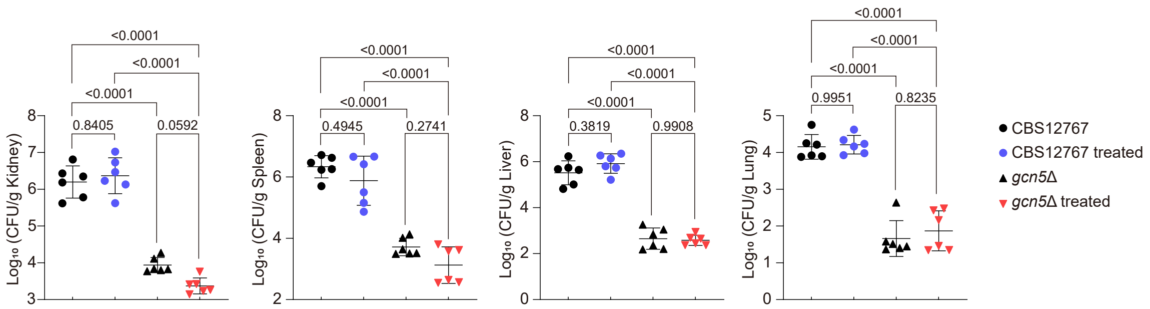

Supplementary Fig. 6 *In vivo* evaluation of fluconazole-mediated clearance against *C. auris gcn5*Δ strain. The fungal burden was determined in the kidneys, spleen, liver, and lungs of mice from each group following 8 doses of treatment with 20 mg/kg FLC or an equivalent volume of solvent (n=6). Data are expressed as mean ± SD. Statistical significance analysis was performed using one-way ANOVA with Sidak's test. Source data are provided as a Source Data file.

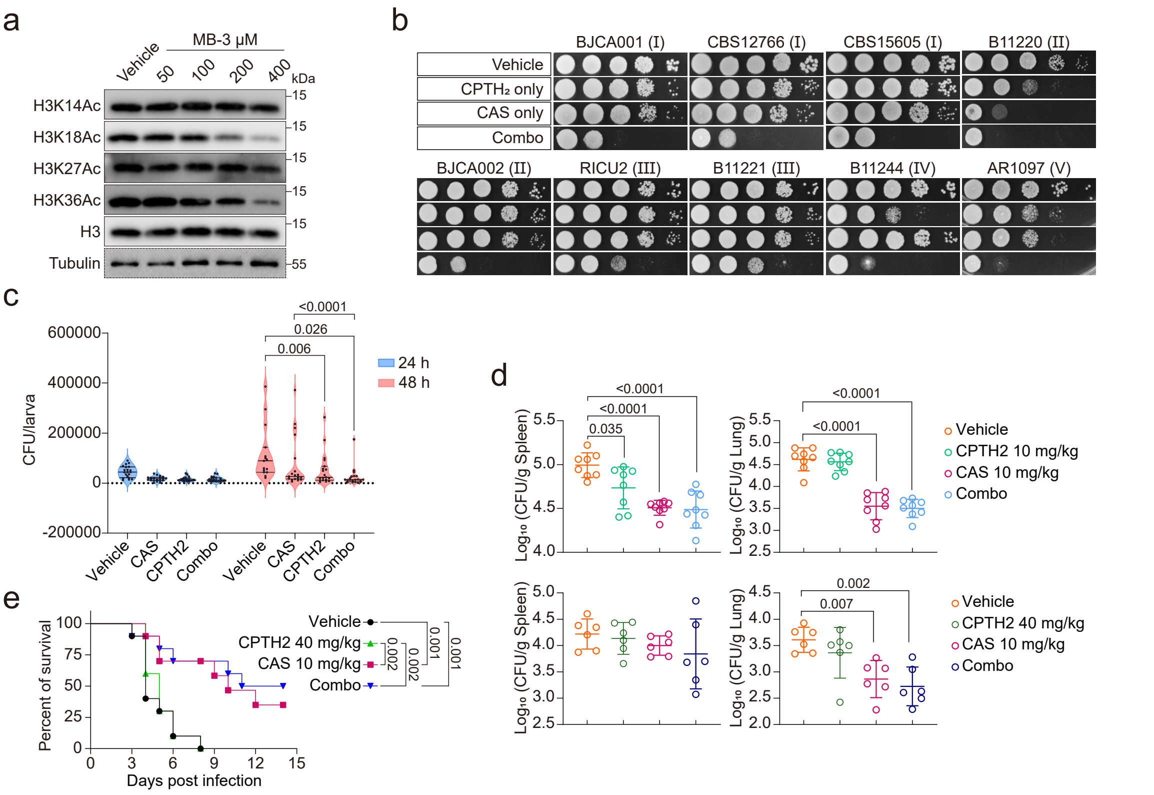

Supplementary Fig. 7 *In vitro* and *in vivo* assessment of Gcn5-targeted inhibition synergizing with CAS against *C. auris*. a Western blot analysis evaluating the inhibitory effect of the Gcn5-specific inhibitor MB-3 on histone H3 acetylation in *C. auris* CBS12767. The experiment was independently repeated twice with consistent results. **b** Spot assay assessing the antifungal effects of $CPTH_2$ and CAS, both alone and in combination, against various clades of *C. auris* (Clades I, II, III, IV, and V). **c** Fungal burden in each *Galleria mellonella* larva infected with 1×10^6 CFU of *C. auris* yCB799 and treated with either CAS (0.5 μg/mL) or $CPTH_2$ (16 ng/mL) alone or in combination, or an equivalent volume of solvent, at 24 and 48 h post-infection (n=20). **d** Fungal burden measured in the spleen and lungs of mice infected with 2×10^7 CFU of *C. auris* yCB799 after 6 treatments with $CPTH_2$ (10 mg/kg, n=6; 40 mg/kg, n=8) and CAS (10 mg/kg), either alone or in combination, or an equivalent volume of solvent. **e** Survival curve of immunosuppressed mice infected with 5×10^7 CFU of *C. auris* yCB799 and treated with six doses of $CPTH_2$ (40 mg/kg) and CAS (10 mg/kg), either alone or in combination, or an equivalent volume of solvent (n=10). Data presented in (**c**, **d)** are expressed as mean ± SD. Statistical significance analysis was performed using Log-rank (Mantel-Cox) test (**e**) or one-way ANOVA with Sidak's test (**c**, **d**). Source data are provided as a Source Data file.

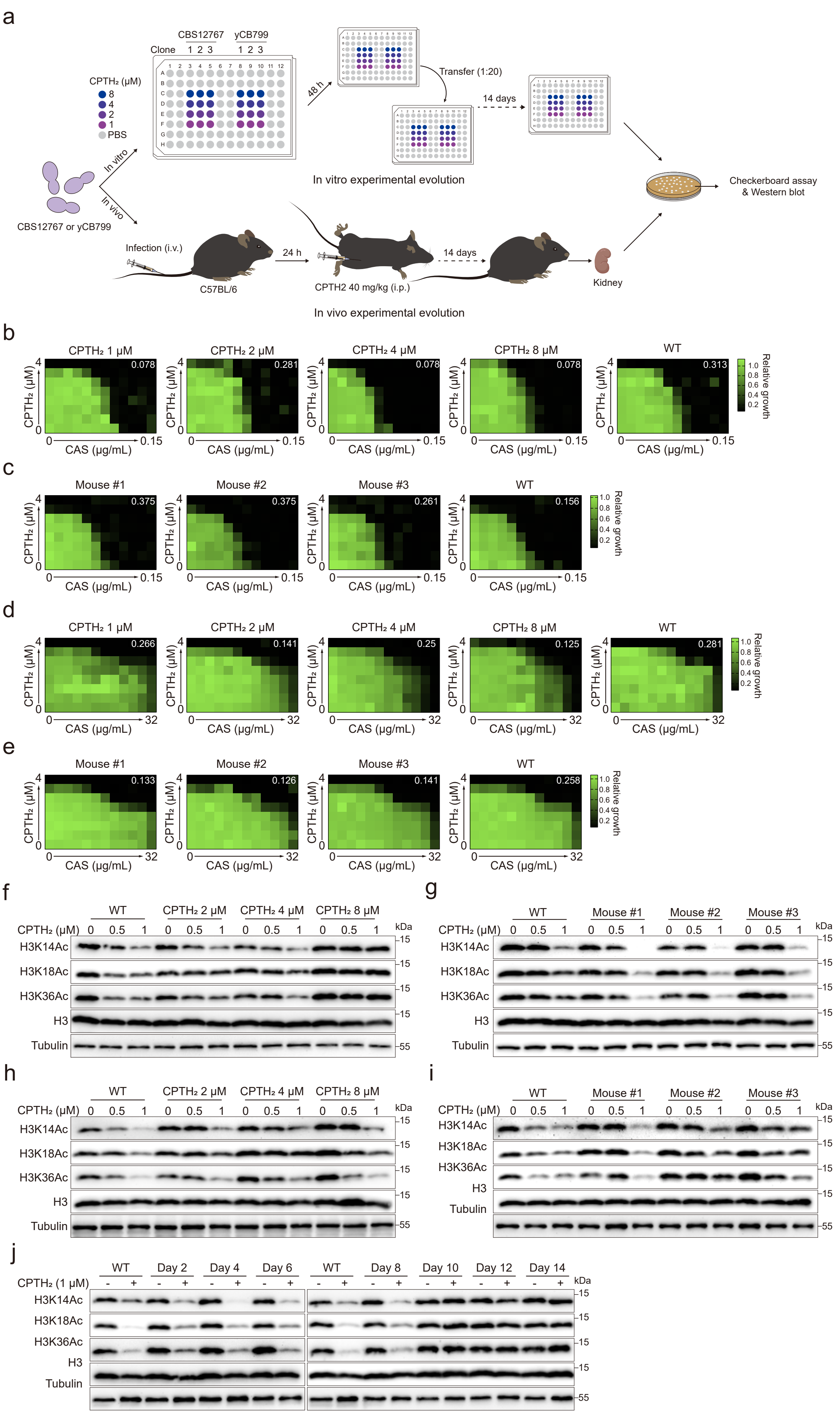

a
CBS12767
yCB799
Clone 1 2 3 1 2 3
CPTH$_2$ (μM)
8
4
2
1
PBS
48 h
Transfer (1:20)
14 days
In vitro experimental evolution
In vitro
In vivo
CBS12767 or yCB799
Checkerboard assay & Western blot
Infection (i.v.)
C57BL/6
24 h
CPTH2 40 mg/kg (i.p.)
14 days
Kidney
In vivo experimental evolution
b
CPTH$_2$ 1 μM
CPTH$_2$ 2 μM
CPTH$_2$ 4 μM
CPTH$_2$ 8 μM
WT
0.078
0.281
0.078
0.078
0.313
CPTH$_2$ (μM)
CAS (μg/mL)
0.15
Relative growth
1.0
0.8
0.6
0.4
0.2
c
Mouse #1
Mouse #2
Mouse #3
WT
0.375
0.375
0.261
0.156
d
CPTH$_2$ 1 μM
CPTH$_2$ 2 μM
CPTH$_2$ 4 μM
CPTH$_2$ 8 μM
WT
0.266
0.141
0.25
0.125
0.281
32
e
Mouse #1
Mouse #2
Mouse #3
WT
0.133
0.126
0.141
0.258
f
WT
CPTH$_2$ 2 μM
CPTH$_2$ 4 μM
CPTH$_2$ 8 μM
CPTH$_2$ (μM) 0 0.5 1 0 0.5 1 0 0.5 1 0 0.5 1
kDa
H3K14Ac
H3K18Ac
H3K36Ac
H3
Tubulin
15
55
g
WT
Mouse #1
Mouse #2
Mouse #3
CPTH$_2$ (μM) 0 0.5 1 0 0.5 1 0 0.5 1 0 0.5 1
H3K14Ac
H3K18Ac
H3K36Ac
H3
Tubulin
h
WT
CPTH$_2$ 2 μM
CPTH$_2$ 4 μM
CPTH$_2$ 8 μM
i
WT
Mouse #1
Mouse #2
Mouse #3
j
WT
Day 2
Day 4
Day 6
WT
Day 8
Day 10
Day 12
Day 14
CPTH$_2$ (1 μM)
- + - + - + - + - + - + - + - + - +
H3K14Ac
H3K18Ac
H3K36Ac
H3
Tubulin

Supplementary Fig. 8 Evolution of resistance in *C. auris* to CPTH$_2$-mediated inhibition of histone H3 acetylation after prolonged exposure, both *in vitro* and *in vivo*. a Schematic of the experimental design for the *in vitro* and *in vivo* evolution assays (n=3). **b**-**e** Checkerboard assay for antifungal combinations. Growth heatmaps showing the effects of CPTH$_2$ and CAS combinations on *C. auris* CBS12767 (**b**, **c**) or yCB799 (**d**, **e**) after 14 days of CPTH$_2$ treatment under *in vitro* (**b**, **d**) and *in vivo* (**c**, **e**) conditions. FICI values are shown in the upper right corners. **f**-i Western blot analysis showing the effect of CPTH$_2$ on histone H3 acetylation in *C. auris* CBS12767 (**f**, **g**) or yCB799 (**h**, **i**) after 14 days of CPTH$_2$ treatment under *in vitro* (**f**, **h**) and *in vivo* (**g**, **i**) conditions. **j** Western blot analysis of histone H3 acetylation in *C. auris* CBS12767 after *in vitro* treatment with 8 μM CPTH$_2$ for different durations. Source data are provided as a Source Data file.

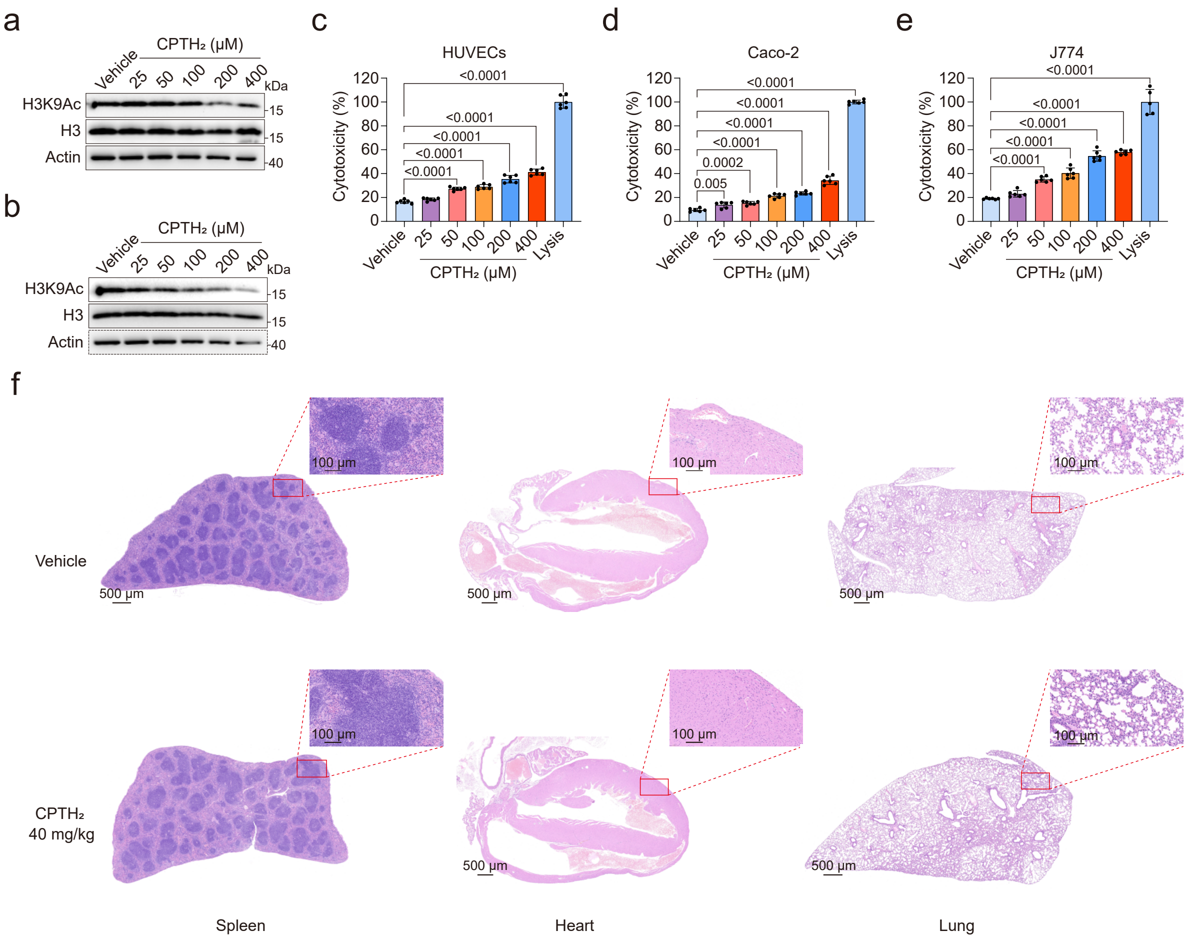

Supplementary Fig. 9 Evaluating the safety of targeting Gcn5 for antifungal therapy against *C. auris* infections. **a-b** Western blot analysis demonstrating the effect of $CPTH_2$ on histone H3K9 acetylation in Caco-2 (**a**) and HUVEC (**b**) cells. Each experiment was independently repeated twice with consistent results. **c-e** Assessment of LDH release in HUVEC (**c**), Caco-2 (**d**), and J774 (**e**) cells treated with varying concentrations of $CPTH_2$ or an equivalent volume of DMSO for 24 hours (n=6). **f** Histological analysis of spleen, heart, and lungs in all 4 mice from each group, with consistent results observed across individuals. Data presented in (**c-e**) are expressed as mean ± SD. Statistical significance analysis was performed using one-way ANOVA with Sidak's test. Source data are provided as a Source Data file.

Supplementary Table 1 MICs of various antifungal agents against *C. auris* strains.

Angifungal agent	Time(h)	MIC(μg/mL)					
		CBS12767	*gcn5Δ*	*rtt109Δ*	*set1Δ*	*Set2Δ*	*dot1Δ*
Caspofungin	48	0.25	0.0625	0.25	0.25	0.25	0.25
Micafungin	48	0.25	0.125	0.125	0.25	0.25	0.25
Fluconazole	24	256	64	128	128	256	256
Voriconazole	24	1	0.125	1	0.5	1	1
Itraconazole	24	1	0.063	1	1	1	1
Amphotericin B	24	2	0.125	2	2	2	2

Supplementary Table 2 FICI values for combinations of CAS and $CPTH_2$ against *C. auris* strains from different clades.

		CAS			CPTH2			FICI
		**MIC $_{alone}$ ng/mL**	**MIC $_{combination}$ ng/mL**	**FIC**	**MIC $_{alone}$ µM/L**	**MIC $_{combination}$ µM/L**	**FIC**	
Clade I	CBS12767	9.375	2.344	0.25	2	0.125	0.063	0.313
	yCB799	> 32000	1000	0.031	2	0.5	0.25	0.281
	BJCA001	4.688	0.586	0.125	2	0.125	0.063	0.188
	CBS12766	18.75	2.344	0.125	2	0.125	0.063	0.188
	CBS15605	9.375	0.586	0.063	4	0.063	0.016	0.079
Clade II	CBS10913	2.344	1.172	0.5	2	1	0.5	1
	CBS12372	2.344	1.172	0.5	2	0.5	0.25	0.75
Clade III	BJCA002	9.375	2.344	0.25	4	0.5	0.125	0.375
	RICU2	9.375	2.344	0.25	4	0.5	0.125	0.375
	B11221	18.75	1.172	0.063	> 4	1	0.25	0.313
Clade IV	B11244	18.75	2.344	0.125	2	0.25	0.125	0.25
Clade V	AR1097	4.688	1.172	0.25	4	1	0.25	0.5

Supplementray Table 3 FICI values for the combination of CAS and $CPTH_2$ against *C. auris* CBS12767 strains evolved under *in vitro* or *in vivo* $CPTH_2$ pressure.

Evolution Experiment (CBS12767)		CAS			$CPTH_2$			FICI
		MIC $_{alone}$ ng/mL	MIC $_{combination}$ ng/mL	FIC	MIC $_{alone}$ μM/L	MIC $_{combination}$ μM/L	FIC	
in vitro								
**$CPTH_2$ (μM)**	**Replicates**							
1	#1	18.75	1.1719	0.0625	4	0.0625	0.0156	0.0781
	#2	9.375	2.3438	0.25	4	0.125	0.0313	0.2813
	#3	9.375	1.1719	0.125	4	1	0.25	0.375
2	#1	9.375	2.3438	0.25	4	0.125	0.0313	0.2813
	#2	4.6875	1.1719	0.25	4	0.125	0.0313	0.2813
	#3	9.375	2.3438	0.25	> 4	1	0.125	0.375
4	#1	9.375	0.5829	0.0625	4	0.0625	0.0156	0.0781
	#2	4.6875	0.5859	0.125	4	0.25	0.0625	0.1875
	#3	4.6875	0.1465	0.0313	> 4	2	0.25	0.2813
8	#1	9.375	0.5859	0.0625	4	0.0625	0.0156	0.0781
	#2	4.6875	0.5859	0.125	4	0.0625	0.0156	0.1406
	#3	18.75	0.5829	0.0313	4	0.0625	0.0156	0.0469
in vivo								
**Mouse**	**Clone**							
#1	#1	4.6875	1.1719	0.25	2	0.25	0.125	0.375
	#2	4.6875	1.1719	0.25	2	0.25	0.125	0.375
	#3	4.6875	1.1719	0.25	2	0.125	0.0625	0.3125
	#4	4.6875	1.1719	0.25	2	0.0625	0.0313	0.2813
	#5	9.375	1.1719	0.125	2	0.125	0.0625	0.1875
	#6	4.6875	1.1719	0.25	2	0.125	0.0625	0.3125
	#7	4.6875	1.1719	0.25	2	0.5	0.25	0.5
	#8	4.6875	0.1465	0.0313	4	0.5	0.125	0.1563
#2	#1	4.6875	1.1719	0.25	2	0.5	0.25	0.5
	#2	4.6875	0.1465	0.0313	4	0.5	0.125	0.1563
	#3	4.6875	1.1719	0.25	2	0.25	0.125	0.375
	#4	4.6875	1.1719	0.25	2	0.25	0.125	0.375
	#5	9.375	1.1719	0.125	2	0.0625	0.0313	0.1563
	#6	4.6875	1.1719	0.25	2	0.25	0.125	0.375
	#7	4.6875	1.1719	0.25	2	0.0625	0.0313	0.2813
	#8	4.6875	1.1719	0.25	2	0.25	0.125	0.375
#3	#1	4.6875	1.1719	0.25	2	0.5	0.25	0.5
	#2	4.6875	1.1719	0.25	2	1	0.5	0.75
	#3	4.6875	1.1719	0.25	2	0.0625	0.0313	0.2813
	#4	4.6875	1.1719	0.25	4	0.25	0.0625	0.3125
	#5	4.6875	1.1719	0.25	2	0.125	0.0625	0.3125
	#6	4.6875	0.1465	0.0313	4	0.25	0.0625	0.0938
	#7	4.6875	1.1719	0.25	2	0.0625	0.0313	0.2813
	#8	4.6875	1.1719	0.25	2	0.125	0.0625	0.3125

Supplementray Table 4 FICI values for the combination of CAS and $CPTH_2$ against *C. auris* yCB799 strains evolved under *in vitro* or *in vivo* $CPTH_2$ pressure

Evolution Experiment (yCB799)		CAS			$CPTH_2$			FICI
		MIC $_{alone}$ μg/mL	MIC $_{combination}$ μg/mL	FIC	MIC $_{alone}$ μM/L	MIC $_{combination}$ μM/L	FIC	
in vitro								
**$CPTH_2$ (μM)**	**Replicates**							
1	#1	32	0.5	0.0156	4	1	0.25	0.2656
	#2	16	1	0.0625	4	0.5	0.125	0.1875
	#3	32	2	0.0625	4	0.25	0.0625	0.125
2	#1	32	0.5	0.0156	4	0.5	0.125	0.1406
	#2	32	1	0.0313	4	0.25	0.0625	0.0938
	#3	32	0.5	0.0156	4	0.5	0.125	0.1406
4	#1	8	1	0.125	4	0.5	0.125	0.25
	#2	8	1	0.125	4	0.5	0.125	0.25
	#3	32	2	0.0625	4	0.5	0.125	0.1875
8	#1	16	1	0.0625	4	0.25	0.0625	0.125
	#2	16	2	0.125	4	0.5	0.125	0.25
	#3	8	2	0.25	4	0.5	0.125	0.375
in vivo								
**Mouse**	**Clone**							
#1	#1	32	0.25	0.0078	4	0.5	0.125	0.1328
	#2	32	0.25	0.0078	4	0.5	0.125	0.1328
	#3	32	1	0.0313	2	0.25	0.125	0.1563
	#4	32	1	0.0313	4	1	0.25	0.2813
	#5	32	0.25	0.0078	4	0.5	0.125	0.1328
	#6	32	0.125	0.0039	4	0.5	0.125	0.1289
	#7	32	0.125	0.0039	4	0.5	0.125	0.1289
	#8	32	1	0.0313	2	0.5	0.25	0.2813
#2	#1	32	0.5	0.0156	4	0.5	0.125	0.1406
	#2	32	1	0.0313	2	0.5	0.25	0.2813
	#3	32	2	0.0625	2	0.5	0.25	0.3125
	#4	32	2	0.0625	2	0.5	0.25	0.3125
	#5	32	1	0.0313	2	0.25	0.125	0.1563
	#6	32	0.125	0.0039	4	0.5	0.125	0.1289
	#7	32	0.5	0.0156	4	0.5	0.125	0.1406
	#8	32	2	0.0625	2	0.5	0.25	0.3125
#3	#1	32	0.5	0.0156	4	0.5	0.125	0.1406
	#2	32	0.5	0.0156	4	0.5	0.125	0.1406
	#3	32	1	0.0313	2	0.25	0.125	0.1563
	#4	32	0.5	0.0156	4	0.5	0.125	0.1406
	#5	32	0.5	0.0156	4	0.5	0.125	0.1406
	#6	32	0.5	0.0156	4	0.5	0.125	0.1406
	#7	32	0.5	0.0156	4	0.5	0.125	0.1406
	#8	32	0.5	0.0156	4	0.5	0.125	0.1406

Supplementary Table 5 Antibodies used in this study

Name	Identifier	Source
Recombinant Anti-Histone H3 (acetyl K9) antibody	ab32129	Abcam
Recombinant Anti-Histone H3 (acetyl K14) antibody	ab52946	Abcam
Recombinant Anti-Histone H3 (acetyl K18) antibody	ab40888	Abcam
Anti-Histone H3 (acetyl K27) antibody	ab4729	Abcam
Recombinant Anti-Histone H3 (acetyl K36) antibody	ab177179	Abcam
Histone H3K56ac antibody (pAb)	39282	Activemotif
Recombinant Anti-Histone H3 (mono methyl K4) antibody	ab176877	Abcam
Recombinant Anti-Histone H3 (di methyl K4) antibody	ab32356	Abcam
Recombinant Anti-Histone H3 (tri methyl K4) antibody	ab313500	Abcam
Recombinant Anti-Histone H3 (mono methyl K36) antibody	ab176920	Abcam
Recombinant Anti-Histone H3 (di methyl K36) antibody	ab176921	Abcam
Anti-Histone H3 (tri methyl K36) antibody	ab9050	Abcam
Recombinant Anti-Histone H3 (tri methyl K79) antibody	ab208189	Abcam
Phospho-p44/42 MAPK (Erk1/2) (Thr202/Tyr204) antibody	9101S	CST
Phospho-p38 MAPK mAb	4511S	CST
Anti-Tubulin antibody	NB100-1639	Novus Biologicals
Normal Rabbit IgG	2729S	CST
