# Supplementary material for: Targeting epigenetic regulators to overcome drug resistance in the emerging human fungal pathogen Candida auris
Source: Nat Commun. 2025 May 20;16:4668. doi: 10.1038/s41467-025-59898-6 (PMC12092656; doi:10.1038/s41467-025-59898-6)
Supplement: Supplementary file 2 — Description Of Additional Supplementary File [file 41467_2025_59898_MOESM2_ESM.pdf]

Description of Additional supplementary files

Supplementary Data 1.

C. auris strains and plasmid used in this study

Supplementary Data 2.

Primers used in this study
