# Supplementary material for: Targeting epigenetic regulators to overcome drug resistance in the emerging human fungal pathogen Candida auris
Source: Nat Commun. 2025 May 20;16:4668. doi: 10.1038/s41467-025-59898-6 (PMC12092656; doi:10.1038/s41467-025-59898-6)
Supplement: Supplementary file 4 — Reporting summary [file 41467_2025_59898_MOESM4_ESM.pdf]

nature portfolio

Corresponding author(s): Changbin Chen, Xiaotian Huang, Dongsheng Zhou

Last updated by author(s): Apr 3, 2025

Reporting Summary

Nature Portfolio wishes to improve the reproducibility of the work that we publish. This form provides structure for consistency and transparency in reporting. For further information on Nature Portfolio policies, see our Editorial Policies and the Editorial Policy Checklist.

Statistics

For all statistical analyses, confirm that the following items are present in the figure legend, table legend, main text, or Methods section.

n/a	Confirmed	
☐	☒	The exact sample size (*n*) for each experimental group/condition, given as a discrete number and unit of measurement
☐	☒	A statement on whether measurements were taken from distinct samples or whether the same sample was measured repeatedly
☐	☒	The statistical test(s) used AND whether they are one- or two-sided<br>*Only common tests should be described solely by name; describe more complex techniques in the Methods section.*
☒	☐	A description of all covariates tested
☐	☒	A description of any assumptions or corrections, such as tests of normality and adjustment for multiple comparisons
☐	☒	A full description of the statistical parameters including central tendency (e.g. means) or other basic estimates (e.g. regression coefficient) AND variation (e.g. standard deviation) or associated estimates of uncertainty (e.g. confidence intervals)
☐	☒	For null hypothesis testing, the test statistic (e.g. *F*, *t*, *r*) with confidence intervals, effect sizes, degrees of freedom and *P* value noted<br>*Give P values as exact values whenever suitable.*
☒	☐	For Bayesian analysis, information on the choice of priors and Markov chain Monte Carlo settings
☒	☐	For hierarchical and complex designs, identification of the appropriate level for tests and full reporting of outcomes
☒	☐	Estimates of effect sizes (e.g. Cohen's *d*, Pearson's *r*), indicating how they were calculated

Our web collection on statistics for biologists contains articles on many of the points above.

Software and code

Policy information about availability of computer code

Data collection	*Provide a description of all commercial, open source and custom code used to collect the data in this study, specifying the version used OR state that no software was used.*
Data analysis	Gray value analysis of immunoblot bands was performed using ImageJ (V2.3.0). Spot assay, Western blot and micrograph images were processed using Photoshop CC V14.0). Graphs and statistical analyses were conducted using GraphPad Prism 9 (V9.4.1). Trim Galore, STAR, DESeq2 and clusterProfiler were used for RNA-Seq analysis.

For manuscripts utilizing custom algorithms or software that are central to the research but not yet described in published literature, software must be made available to editors and reviewers. We strongly encourage code deposition in a community repository (e.g. GitHub). See the Nature Portfolio guidelines for submitting code & software for further information.

Data

Policy information about availability of data

All manuscripts must include a data availability statement. This statement should provide the following information, where applicable:

- Accession codes, unique identifiers, or web links for publicly available datasets
- A description of any restrictions on data availability
- For clinical datasets or third party data, please ensure that the statement adheres to our policy

The authors declare that all data supporting the findings of this study are available within the article and its Supplementary Information files. RNA-Seq data can be found under the GEO accession number GSE293594. Source data are provided in this paper.

Research involving human participants, their data, or biological material

Policy information about studies with human participants or human data. See also policy information about sex, gender (identity/presentation), and sexual orientation and race, ethnicity and racism.

Reporting on sex and gender	*Use the terms sex (biological attribute) and gender (shaped by social and cultural circumstances) carefully in order to avoid confusing both terms. Indicate if findings apply to only one sex or gender; describe whether sex and gender were considered in study design; whether sex and/or gender was determined based on self-reporting or assigned and methods used. Provide in the source data disaggregated sex and gender data, where this information has been collected, and if consent has been obtained for sharing of individual-level data; provide overall numbers in this Reporting Summary. Please state if this information has not been collected. Report sex- and gender-based analyses where performed, justify reasons for lack of sex- and gender-based analysis.*
Reporting on race, ethnicity, or other socially relevant groupings	*Please specify the socially constructed or socially relevant categorization variable(s) used in your manuscript and explain why they were used. Please note that such variables should not be used as proxies for other socially constructed/relevant variables (for example, race or ethnicity should not be used as a proxy for socioeconomic status). Provide clear definitions of the relevant terms used, how they were provided (by the participants/respondents, the researchers, or third parties), and the method(s) used to classify people into the different categories (e.g. self-report, census or administrative data, social media data, etc.) Please provide details about how you controlled for confounding variables in your analyses.*
Population characteristics	*Describe the covariate-relevant population characteristics of the human research participants (e.g. age, genotypic information, past and current diagnosis and treatment categories). If you filled out the behavioural & social sciences study design questions and have nothing to add here, write "See above."*
Recruitment	*Describe how participants were recruited. Outline any potential self-selection bias or other biases that may be present and how these are likely to impact results.*
Ethics oversight	*Identify the organization(s) that approved the study protocol.*

Note that full information on the approval of the study protocol must also be provided in the manuscript.

Field-specific reporting

Please select the one below that is the best fit for your research. If you are not sure, read the appropriate sections before making your selection.

☒ Life sciences ☐ Behavioural & social sciences ☐ Ecological, evolutionary & environmental sciences

For a reference copy of the document with all sections, see nature.com/documents/nr-reporting-summary-flat.pdf

Life sciences study design

All studies must disclose on these points even when the disclosure is negative.

Sample size	No statistical methods were used to predetermine sample size. The sample sizes for the mouse experiments in this study were determined based on our previous related research (Liu et al. Translational Research, 2022) and similar studies published in high-impact journals (Kali R. Iyer et al. Nature communications, 2020; Zhang et al. Science, 2020), ensuring sufficient statistical power.
Data exclusions	In animal experiments (Fig. 6b), mice that died before the sampling time points were excluded. Apart from these cases, no other data were excluded.
Replication	All experiments are replicated at least twice with the same conclusion.
Randomization	Samples/animals were randomly assigned to experimental groups and included in the experiments
Blinding	The investigators were not blinded to allocation during experiments and outcome assessment. The perceived risk of detection/performance bias was deemed negligible.

Reporting for specific materials, systems and methods

We require information from authors about some types of materials, experimental systems and methods used in many studies. Here, indicate whether each material, system or method listed is relevant to your study. If you are not sure if a list item applies to your research, read the appropriate section before selecting a response.

Materials & experimental systems

n/a	Involved in the study
☐	☒ Antibodies
☐	☒ Eukaryotic cell lines
☒	☐ Palaeontology and archaeology
☐	☒ Animals and other organisms
☒	☐ Clinical data
☒	☐ Dual use research of concern
☒	☐ Plants

Methods

n/a	Involved in the study
☒	☐ ChIP-seq
☒	☐ Flow cytometry
☒	☐ MRI-based neuroimaging

Antibodies

Antibodies used

1. Recombinant Anti-Histone H3 (acetyl K9) antibody (Abcam, ab32129) used at 1:1000
2. Recombinant Anti-Histone H3 (acetyl K14) antibody (Abcam, ab52946) used at 1:1000
3. Recombinant Anti-Histone H3 (acetyl K18) antibody (Abcam, ab40888) used at 1:1000
4. Anti-Histone H3 (acetyl K27) antibody (Abcam, ab4729) used at 1:1000
5. Recombinant Anti-Histone H3 (acetyl K36) antibody (Abcam, ab177179) used at 1:1000
6. Histone H3K56ac antibody (pAb) (Activemotif, 39282) used at 1:1000
7. Recombinant Anti-Histone H3 (mono methyl K4) antibody (Abcam, ab176877) used at 1:1000
8. Recombinant Anti-Histone H3 (di methyl K4) antibody, (Abcam, ab32356) used at 1:1000
9 . Recombinant Anti-Histone H3 (tri methyl K4) antibody, (Abcam, ab313500) used at 1:1000
10. Recombinant Anti-Histone H3 (mono methyl K36) antibody, (Abcam, ab176920) used at 1:1000
11. Recombinant Anti-Histone H3 (di methyl K36) antibody, (Abcam, ab176921) used at 1:1000
12. Anti-Histone H3 (tri methyl K36) antibody, (Abcam, ab9050) used at 1:1000
13. Recombinant Anti-Histone H3 (tri methyl K79) antibody, (Abcam, ab208189) used at 1:1000
14. Phospho-p44/42 MAPK (Erk1/2) (Thr202/Tyr204) antibody, (CST, 9101S) used at 1:1000
15. Phospho-p38 MAPK mAb (CST, 4511S) used at 1:1000
16. Normal Rabbit IgG (CST, 2729S) used at 1:1000
17. Anti-Tubulin antibody (Novus Biologicals, NB100-1639), used at 1:2000

Validation

1. https://www.abcam.com/en-us/products/primary-antibodies/histone-h3-acetyl-k9-antibody-y28-chip-grade-ab32129
2. https://www.abcam.com/en-us/products/primary-antibodies/histone-h3-acetyl-k14-antibody-ep964y-chip-grade-ab52946
3. https://www.abcam.com/en-us/products/primary-antibodies/histone-h3-acetyl-k18-antibody-ep959y-chip-grade-ab40888
4. https://www.abcam.com/en-us/products/primary-antibodies/histone-h3-acetyl-k27-antibody-chip-grade-ab4729
5. https://www.abcam.com/en-us/products/primary-antibodies/histone-h3-acetyl-k36-antibody-epr16992-ab177179
6. https://www.activemotif.com/catalog/details/39281/histone-h3-acetyl-lys56-antibody-pab
7. https://www.abcam.com/en-us/products/primary-antibodies/histone-h3-mono-methyl-k4-antibody-erp16597-chip-grade-ab176877
8. https://www.abcam.com/en-us/products/primary-antibodies/histone-h3-di-methyl-k4-antibody-y47-chip-grade-ab32356
9. https://www.abcam.com/en-us/products/primary-antibodies/histone-h3-tri-methyl-k4-antibody-epr20551-225-chip-grade-bsa-and-azide-free-ab313500
10. https://www.abcam.com/en-us/products/primary-antibodies/histone-h3-mono-methyl-k36-antibody-epr16993-ab176920
11. https://www.abcam.com/en-us/products/primary-antibodies/histone-h3-di-methyl-k36-antibody-epr169942-chip-grade-ab176921
12. https://www.abcam.com/en-us/products/primary-antibodies/histone-h3-tri-methyl-k36-antibody-chip-grade-ab9050
13. https://www.abcam.com/en-us/products/primary-antibodies/histone-h3-tri-methyl-k79-antibody-epr174682-chip-grade-ab208189
14. https://www.cellsignal.com/products/primary-antibodies/phospho-p44-42-mapk-erk1-2-thr202-tyr204-antibody/9101
15. https://www.cellsignal.com/products/primary-antibodies/phospho-p38-mapk-thr180-tyr182-d3f9-xp-rabbit-mab/4511
16. https://www.cellsignal.com/products/primary-antibodies/normal-rabbit-igg/2729
17. https://www.novusbio.com/products/alpha-tubulin-antibody-yol1-34_nb100-1639
srsltid=AfmBOormOA3xSDWSMQXIZEp3x1NUAymvq3vTmR9FVRHbSwdB5skycbru

Eukaryotic cell lines

Policy information about cell lines and Sex and Gender in Research

Cell line source(s)

All cell lines used in this study (HeLa, ATCC CCL-2; Caco-2, ATCC HTB-37; HUVEC, ATCC CRL-1730; J774A.1, ATCC TIB-67) were obtained from the American Type Culture Collection (ATCC).

Authentication	Cell lines used were not authenticated.
Mycoplasma contamination	Cell lines were not tested for mycoplasma contamination, but no signs of contamination were observed.
Commonly misidentified lines (See ICLAC register)	No commonly misidentified cell lines were used.

Animals and other research organisms

Policy information about studies involving animals; ARRIVE guidelines recommended for reporting animal research, and Sex and Gender in Research

Laboratory animals	Female ICR mice (6-8 weeks old, weighing 24-26 g) and female C57BL/6 mice (6-8 weeks old, weighing 18-20 g) were procured from Beijing Vital River Laboratory Animal Technology Company (Beijing, China). The mice were housed in a pathogen-free environment maintained at 210C with 50-70% relative humidity and a 12-hour light/dark cycle. They were provided with unrestricted access to food and water throughout the study.
Wild animals	No wild animals were used in these studies
Reporting on sex	The study did not involve sex-based analysis.
Field-collected samples	No field collected samples were used in these studies
Ethics oversight	The study protocol received thorough review and approval from the Institutional Animal Care and Use Committee (IACUC) at the Shanghai Institute of Immunity and Infection, Chinese Academy of Sciences (Permit Number: A2023024).

Note that full information on the approval of the study protocol must also be provided in the manuscript.

Plants

Seed stocks	*Report on the source of all seed stocks or other plant material used. If applicable, state the seed stock centre and catalogue number. If plant specimens were collected from the field, describe the collection location, date and sampling procedures.*
Novel plant genotypes	*Describe the methods by which all novel plant genotypes were produced. This includes those generated by transgenic approaches, gene editing, chemical/radiation-based mutagenesis and hybridization. For transgenic lines, describe the transformation method, the number of independent lines analyzed and the generation upon which experiments were performed. For gene-edited lines, describe the editor used, the endogenous sequence targeted for editing, the targeting guide RNA sequence (if applicable) and how the editor was applied.*
Authentication	*Describe any authentication procedures for each seed stock used or novel genotype generated. Describe any experiments used to assess the effect of a mutation and, where applicable, how potential secondary effects (e.g. second site T-DNA insertions, mosiacism, off-target gene editing) were examined.*
